# Effect of skin color on optical properties and the implications for medical optical technologies: a review

**DOI:** 10.1117/1.JBO.29.1.010901

**Published:** 2024-01-24

**Authors:** Kerry Setchfield, Alistair Gorman, A. Hamish R. W. Simpson, Michael G. Somekh, Amanda J. Wright

**Affiliations:** a University of Nottingham, Faculty of Engineering, Optics and Photonics Research Group, Nottingham, United Kingdom; b University of Edinburgh, School of Engineering, Edinburgh, United Kingdom; c University of Edinburgh, Department of Orthopaedics, Division of Clinical and Surgical Sciences, Edinburgh, United Kingdom; d Zhejiang Lab, Hangzhou, China

**Keywords:** optical imaging, absorption and scattering coefficients, transmission, skin, Fitzpatrick skin type scale, racial bias, optical coherence tomography, photodynamic therapy, medical wearables

## Abstract

**Significance:**

Skin color affects light penetration leading to differences in its absorption and scattering properties. COVID-19 highlighted the importance of understanding of the interaction of light with different skin types, e.g., pulse oximetry (PO) unreliably determined oxygen saturation levels in people from Black and ethnic minority backgrounds. Furthermore, with increased use of other medical wearables using light to provide disease information and photodynamic therapies to treat skin cancers, a thorough understanding of the effect skin color has on light is important for reducing healthcare disparities.

**Aim:**

The aim of this work is to perform a thorough review on the effect of skin color on optical properties and the implication of variation on optical medical technologies.

**Approach:**

Published *in vivo* optical coefficients associated with different skin colors were collated and their effects on optical penetration depth and transport mean free path (TMFP) assessed.

**Results:**

Variation among reported values is significant. We show that absorption coefficients for dark skin are ∼6% to 74% greater than for light skin in the 400 to 1000 nm spectrum. Beyond 600 nm, the TMFP for light skin is greater than for dark skin. Maximum transmission for all skin types was beyond 940 nm in this spectrum. There are significant losses of light with increasing skin depth; in this spectrum, depending upon Fitzpatrick skin type (FST), on average 14% to 18% of light is lost by a depth of 0.1 mm compared with 90% to 97% of the remaining light being lost by a depth of 1.93 mm.

**Conclusions:**

Current published data suggest that at wavelengths beyond 940 nm light transmission is greatest for all FSTs. Data beyond 1000 nm are minimal and further study is required. It is possible that the amount of light transmitted through skin for all skin colors will converge with increasing wavelength enabling optical medical technologies to become independent of skin color.

## 1 Introduction

Optical technologies have transformed medical diagnostics, surgery, and therapeutics in recent decades and the medical device industry provides vast opportunity for optical technology. Optical fields in medicine account for $73 billion of the global market and include ophthalmic optics, robotics, lasers, optical surgery, microscopy, and endoscopy, making it the largest technology sector in medicine.^1^ Given the size of this sector, it is likely that there will be advances in optical fibers, making them thinner and smaller; use of imaging techniques such as optical coherence tomography (OCT) will become more widespread with greater resolution and providing noninvasive imaging; and laser-based therapeutics will become more accurate and widespread.^2^ Optical devices are used by the majority of medical disciplines and do not consist only of microscopes; they range from otoscopes and ophthalmoscopes to endoscopes and colonoscopes, including surgical microscopes and imaging during robotically assisted surgery.

**Fig. 1:**
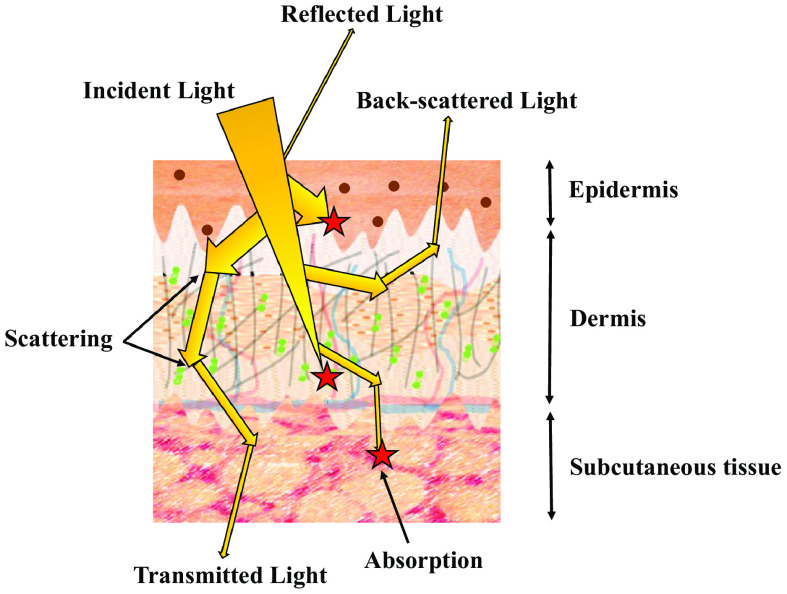
Schematic showing the pathways photons can take when travelling through the skin. Absorption is depicted as red stars; brown spots in the epidermis represent melanin; in the dermis blood vessels are represented by blue and red lines, collagen fibers by gray lines, and elastin by green dots.

Medical optical devices require the interaction of light with human tissue. This interaction can be used to determine the condition of the tissue for analysis or diagnostics, or alternatively it can be used for therapeutics and create changes within the tissue.^2^ Light transmission through the skin is affected by the optical properties of the skin including the absorption and scattering coefficients at given wavelengths as shown in Fig. 1.

Absorption leads to the attenuation of light, and when a photon is absorbed by a molecule (for example by melanin in the epidermis) the energy from the photon is transferred to the molecule and transformed to heat. Scattering events occur when a photon’s direction is changed by a scattering center in the sample (such as collagen). Importantly, the angle of this scatter is taken into account using the anisotropy factor (the average cosine of the scattering angle) and when considered with the mean distance between scattering events, to produce the reduced scattering coefficient, it provides a more useful understanding of how light travels through the skin. The absorption and reduced scattering properties of tissues affect the depth at which light can form a focus, and hence make a measurement or record an image, and also the power (fluence) of light required for photodynamic therapies.^3^ Therefore, development of noninvasive medical devices and therapeutics that use light and optics requires knowledge of the optical properties of tissues and how these properties vary with skin color.

Why is it important to consider skin color? In addition to the long known increased epidermal risk to patients with dark skin by laser therapies such as laser absorption leading to burns, postinflammatory hyperpigmentation, and hypopigmentation due to melanocyte destruction,^4^^–^^6^ the penetration depth of light for imaging purposes and the results from medical wearable devices are affected by skin color.^7^^–^^10^ A recent publication has shown the importance of acquiring data regarding the optical properties of skin of all skin colors for disease monitoring.^11^ It showed that PO, which is used to measure oxygen saturation in the blood, consistently overestimated oxygen levels, and this was particularly significant for darker skin. Pulse oximeters use light of wavelengths 660 and 940 nm to monitor the health of people with diseases such as chronic obstructive pulmonary disease, asthma, pneumonia, lung cancer, anemia, myocardial infarction, and heart failure. This overestimation in oxygen levels had potentially serious side effects in the case of COVID-19 where ethnic groups were more severely affected by infection. Therefore, optical property measurements need to give comparable results for all skin colors to ensure all groups are given equitable healthcare.

**Fig. 2:**
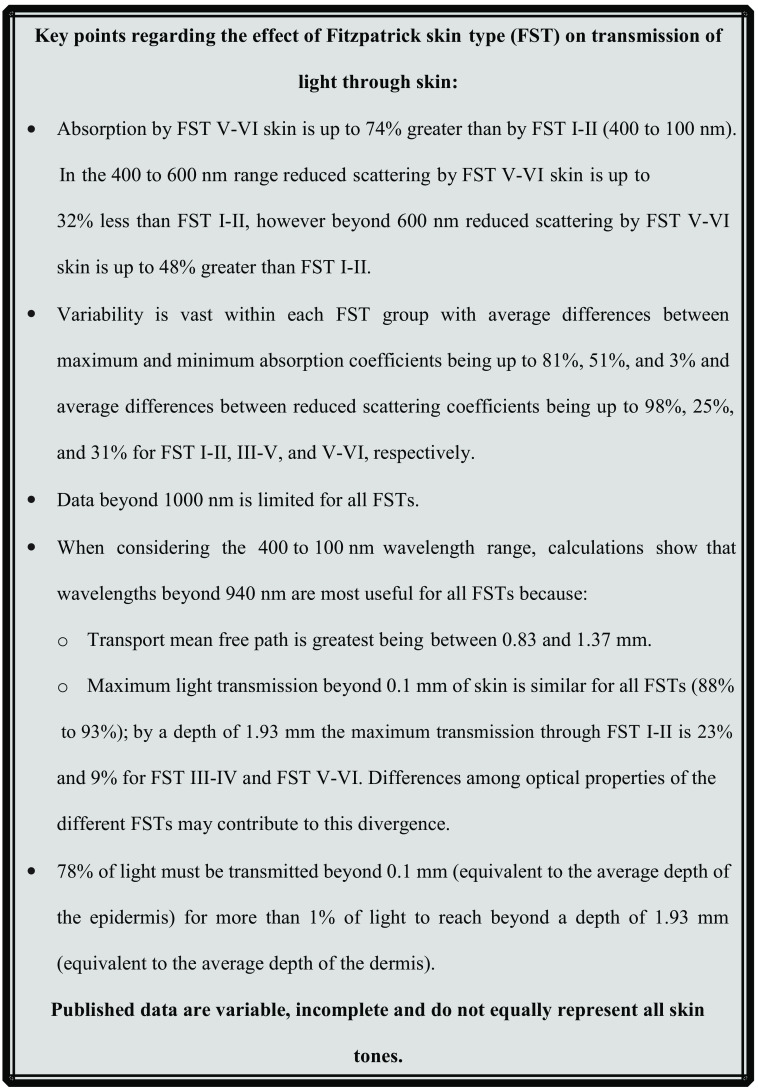
The effect of FST on transmission of light through skin.

**Fig. 3:**
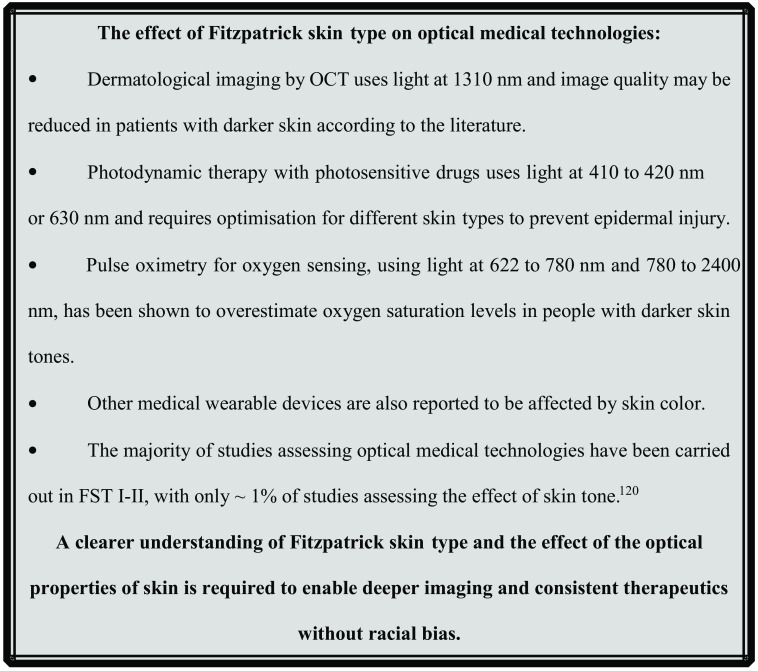
The effect of FST on commonly used optical medical technologies.

The aim of this review is to determine how the optical properties of skin vary with skin color and the implications of any variations on optical medical devices. A thorough literature review is presented collating previously published measurements and identifying regions in the optical spectrum where variation in properties with skin color is minimal and maximal as summarized in Fig. 2. Three case studies are presented assessing the impact of these variations on well-established medical technologies covering OCT, photodynamic therapy (PDT), and medical wearables such as pulse oximeters; the effect of skin color, described by the Fitzpatrick skin type (FST) scale, on these technologies is summarized in Fig. 3.

## 2 Skin Type: Fitzpatrick Scale and Melanin

The FST scale was developed in 1975 by American dermatologist Thomas B. Fitzpatrick to help to understand ultraviolet (UV) dosing for the treatment of skin diseases, including psoriasis. Using the response of an individual’s skin to UV light based on how they tan or burn, a skin type score is given and individuals are classified into one of six skin-phototypes (FST I–VI; Table 1).^12^ The FST scale has been found to be unreliable for darker skin, not taking into account skin color ranges nor accurately providing skin cancer risk, ultimately leading to healthcare disparities.^13^^,^^14^ It is important to note that, although linked to the amount of melanin in the skin, the FST does not quantify concentrations of melanin in the epidermis. Due to the variable interpretation of the Fitzpatrick scale, many alternatives have been suggested. The Lancer ethnicity scale has been suggested for laser treatment in cosmetic surgery.^15^ This takes into account a patient’s ethnic history, which may be different from the individual’s Fitzpatrick scale presentation and has an effect on their reaction to therapy. Other skin color scales that have been used include the Von Luschan’s chromatic scale, which uses color comparison of a region of skin that has not been photo-exposed with a range of 36 opaque glass tiles, and the Roberts scale, which takes other scales into consideration to predict response to trauma from laser therapy or surgery.^16^^–^^18^ However, these alternatives are also subjective, and spectrophotometry may provide a more accurate determination of skin color and tone. Objective measurements can be made of the skin color volume based on colorimetric values measured by spectrophotometry or tri-stimulus colorimetry combined with individual typology angle.^19^ Data relating to the intensity of light reflected from the skin are used to classify skin into six groups ranging from very light to dark and each color group correlated with melanin content. These measurements are less subjective than the FST scale because they estimate the melanin content of skin and distinguish between skin darkening due to increased melanin or increased hemoglobin but have yet to be used extensively by dermatologists due to cost.^20^^,^^21^ However, although nearly 50 years old, and shown to be unreliable, the FST continues to be used by dermatologists today and is recognized as the “gold standard” for classifying skin types.^20^

**Table 1:** Classification of skin pigmentation using the Fitzpatrick scale.

FST	Characteristics associated with skin type
I	Always burns, never tans (pale white skin)
II	Usually burns, tans minimally (white skin)
III	Sometimes burns mildly, tans gradually (light brown skin)
IV	Burns minimally, always tans well (medium brown skin)
V	Very rarely burns, tans very easily (dark brown skin)
VI	Never burns (deeply pigmented dark brown to black skin)

Melanin in the skin is a mix of eumelanin (a brown/black pigment) and pheomelanin (yellow/reddish pigment).^22^ It is produced by melanocytes at the basal layer of the epidermis and stored in either eumelanosomes or pheomelanosomes and transferred to, and internalized by, keratinocytes, which then disseminate throughout the epidermis.^23^^,^^24^ Skin color is determined by the ratio of eumelanin to pheomelanin and the accumulation of eu- and pheomelanosomes within melanocytes in the epidermis.^25^ Hani *et al*.^26^ measured the ratio of eumelanin to pheomelanin in the epidermis of the forearm from 30 individuals of skin type IV using diffuse reflectance spectroscopy and showed that eumelanin absorption was most important when determining the overall optical properties of the skin.

Scattering and absorption in the skin tend to decrease with increasing wavelength.^27^^–^^29^ In the epidermis and dermis, decreasing absorption is due to the steady decrease in melanin and hemoglobin absorption, respectively. The decreasing contribution of Rayleigh scattering is likely to be associated with the steady decrease in scattering coefficients with increasing wavelength.^29^ In the visible spectrum, melanin absorption has the most attenuating effect on light compared with other skin chromophores such as hemoglobin, water, and lipid.^30^ Although melanin absorption is at its peak at visible wavelengths with absorption increasing exponentially at shorter wavelengths in the 300 to 600 nm range, its absorption spectrum is very broad and it contributes to absorption, albeit to a lesser extent, in the infrared spectrum.^4^^,^^31^^,^^32^

Melanin is highly light absorbing in the visible spectrum; however, melanosomes are involved in light scattering in the 600 to 700 nm range due to their high refractive index.^28^ Despite melanin being a strong absorber, the melanosome scattering coefficient is an order of magnitude greater than the absorption coefficient across the 400 to 1600 nm spectrum.^27^^,^^28^ All skin types tend to have the same numbers of melanocytes but the numbers of melanosomes within each and their ability to pass these on to keratinocytes is varied, with more highly pigmented skin having more melanosomes and a greater ability to transfer melanin.^33^ The size of melanosomes also varies among the epithelia of different skin types. Melanosomes in more pigmented skin types tend to be larger (∼1  μm) and dispersed singly, whereas the melanosomes in less pigmented skin tend to be aggregated and smaller (∼0.5  μm).^34^ Clustered melanosomes tend to be approximately half the size of individual melanosomes.^35^ These sizes are comparable with the wavelengths used in many optical medical devices, impacting the type of scattering that will occur.

## 3 Effect of FST on the Published Absorption and Scattering Data

We identified 20 publications that report the *in vivo* absorption and reduced scattering coefficients of the skin. These have given rise to many further papers that rely on the published data to model the behavior of light in the skin. Of the 20 papers reporting the original measured data, only 12 state the FST and of these 7 measure optical properties for FST I–II, 7 measure FST III–IV, and 3 measure FST V–VI. Sample numbers for each study vary widely from 1 to 1734, with the modal sample number being 6. We have previously shown that *in vivo* published data show high inter- and intrapublication variability with differences among the published absorption data being up to 9.6- and 2-fold for reported scattering data.^36^ Here, we pool the data from the 12 papers measuring optical properties with respect to FST and average coefficients determined for three FST groupings: FST I–II, III–IV, and V–VI. Because of the variation among the published data, some authors have attempted to control for various factors such as measurement method, measurement location, and optical property determination model among the published data; however, even among these, variation is still broad. Therefore, the data collated for this study were only separated based on reported FST and location (where stated in the literature; 25% do not quote location) to ascertain trends and differences. As far as possible, the data shown in this review were taken from the dorsal forearm to increase consistency; however, to obtain enough data for comparison, this was not always possible.

### 3.1 Variability Among Absorption and Scattering Coefficients: Individual Publications

**Fig. 4:**
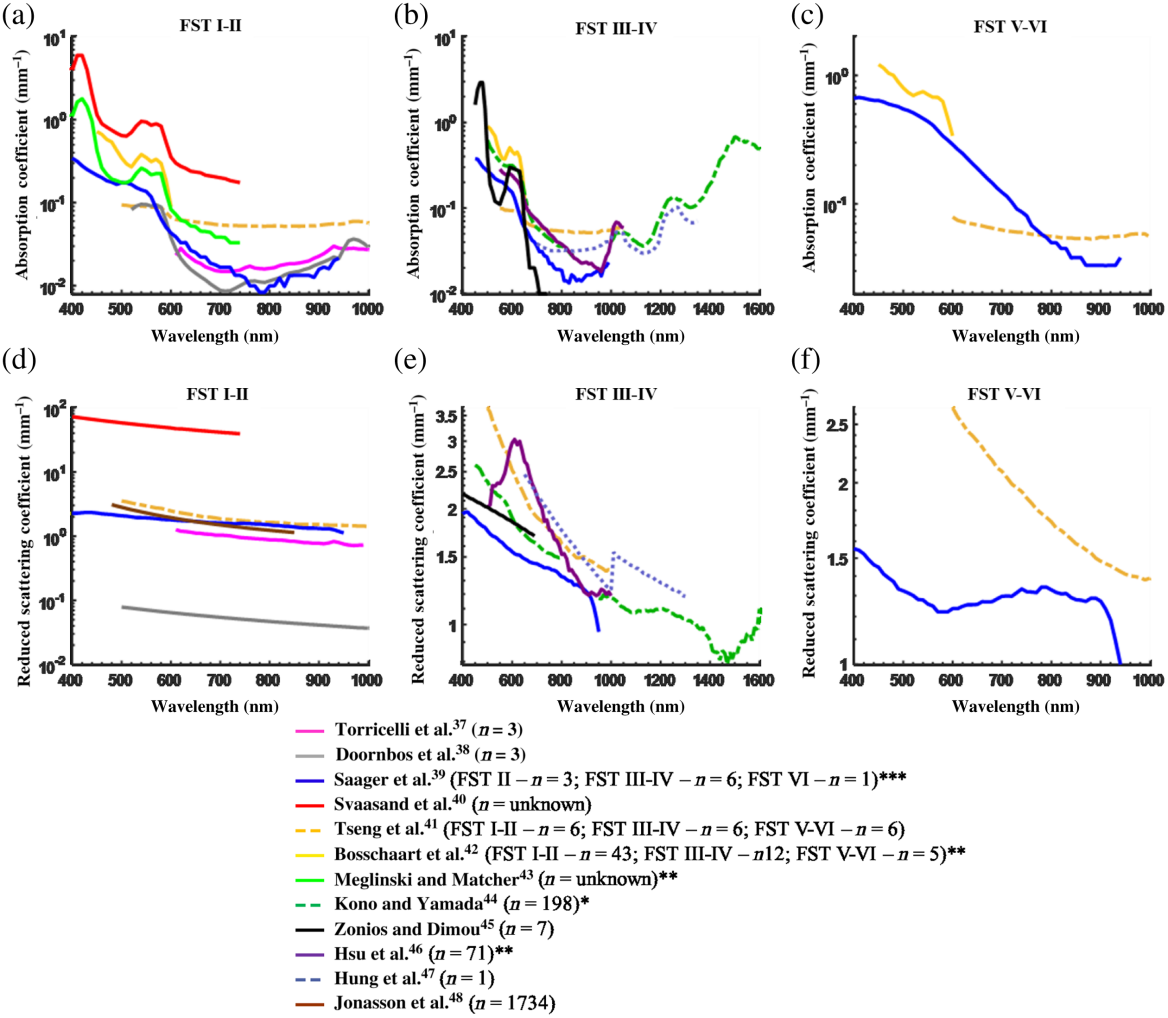
Variability in absorption coefficients (a)–(c) and reduced scattering coefficients (d)–(f) among the published *in vivo* data with skin type. The number of subjects measured for each publication is shown as “n.” *Note that the data from Kono and Yamada^44^ were converted to reduced scattering using an average g taken from the publications of 0.84. **Data from forearm unavailable. ***Note that data measured by Saager et al.^39^ for FST III and FST IV have been averaged to provide an FST III–IV measurement. Due to the reduced scattering scale distortion introduced by outliers, we point out that the distribution of reduced scattering data from Saager et al.^39^ ranges from 2.27 to $1.18\;{\mathrm{mm}}^{-1}$, 1.94 to $1.042\;{\mathrm{mm}}^{-1}$, and 1.56 to $1\;{\mathrm{mm}}^{-1}$ for FST I–II, III–IV, and V–VI, respectively.

The graphs in Fig. 4 show the variability among the published absorption and reduced scattering coefficients for the different FST groups compiled from 12 different publications. As well as the range in spectra used to determine the optical properties of skin, these data also show that there is variability in sample number among the published *in vivo* data and that data are limited for FST V–VI. In total, absorption measurements were taken from in excess of 58 subjects for FST I–II (two papers do not state the sample number) with a modal sample number of 3.^37^^–^^43^ For FST III–IV, there were a total 301 subjects with one paper contributing data measured on 198 recruits and another measured 71 recruits.^39^^,^^41^^,^^42^^,^^44^^–^^47^ The modal subject number was 6. Only three papers detailed the optical properties of FST V–VI from a total of 12 subjects and the average sample number was 4.^39^^,^^41^^,^^42^ Scattering measurements for FST I–II were from in excess of 1749 subjects, with one publication contributing data from 1734 subjects.^37^^–^^41^^,^^48^ Published scattering data were available from 289 subjects for FST III–IV.^39^^,^^41^^,^^44^^–^^47^ Only two manuscripts described scattering data for FST V–VI from a total of seven recruits.^39^^,^^41^

Variability between the published data within each FST group at given wavelength is huge with the average difference between the maximum and minimum reduced scattering coefficients across the measured wavelengths being 98%, 25%, and 31% for FST I–II, III–V, and V–VI, respectively. Average differences between the maximum and minimum reported absorption coefficients were 81%, 51%, and 38% for FST I–II, III–V, and V–VI, respectively.

Each author took measurements in different spectral bands, making reliable comparisons between the data difficult. The differences and trends among the publications are discussed here in further detail.

Tseng et al.^41^ split subjects into three skin type groups of six subjects based on melanin content using diffuse reflectance spectroscopy took measurements in the wavelength range 500 to 1000 nm. A decrease in absorption as the wavelength increased from 500 to 600 nm of twofold was observed for both skin types I–II and III–IV. The optical properties of FST V–VI were not measured in this region because this skin type was too absorbing for their measurement method. The mean absorption coefficients for FST III–IV were greater between 500 and 600 nm (the region coinciding with high melanin absorption) than for FST I–II. In the 600 to 800 nm spectrum, scattering by FST V–VI was greater than the other FSTs. Tseng et al.^41^ suggested that, since melanosomes (up to 1.44  μm in diameter^34^^,^^35^) scatter light and scattering power is related to dimensions of the scatterer, the density of these in FST V–VI may contribute to increased scattering measurements observed in this study among this group.^41^

Measurements by Tseng et al.^41^ show that in the near-infrared (NIR) region of the spectrum, FSTs I–II and III–IV had very similar absorption and scattering properties. For FST V–VI absorption and scattering converged with the other skin types at 850 and 750 nm, respectively, indicating that the NIR is likely to be the region where FST may have less impact on optical properties. The differences between skin types I–II and V–VI were shown to be significant for both absorption and scattering in 600 to 850 nm and 600 to 750 nm ranges, respectively. A small peak in absorption was observed at ∼970  nm for all skin types corresponding with water absorption.

Bosschaart et al.^42^ took measurements in the visible spectrum (400 to 600 nm) of skin from neonates aged between 6 and 28 days with varying skin pigmentation at four different locations. In neonates, skin location did not have a significant effect on optical coefficients, but absorption was shown to increase significantly in skin type V–VI compared with skin type I–II and III–IV patients with time after birth. The data shown in Fig. 4 are taken from the median values for all measurements taken independent of time after birth (as shown in figure 7 from Ref. 42). Coefficients ranged among the grouped skin types between 0.02 to $1.25\;{\mathrm{mm}}^{-1}$ for absorption and 1 to $2.8\;{\mathrm{mm}}^{-1}$ for scattering.

Although the data gathered by both Bosschaart et al.^42^ and Tseng et al.^41^ only overlapped in the 500 to 600 nm spectrum, the variability in resulting data is exemplified by coefficient differences for each skin type in this region. At 600 nm, absorption data published by Bosschaart et al.^42^ are progressively greater with increasing skin pigmentation than Tseng et al.’s^41^ data, being 25%, 45%, and 77% greater for FST I–II, III–IV, and V–VI, respectively. At 500 nm, the differences are more marked, with Bosschaart et al.’s^42^ data being 73% and 77% greater than Tseng et al.’s^41^ for FST I–II and III–IV, respectively.

To quantify melanin in the epidermis of different FSTs, Saager et al.^39^ measured the optical properties of the dorsal forearm for 12 subjects ranging in skin type and including FSTs I, II, III, IV, V, and VI where the number of subjects for each was 1, 3, 5, 1, 1, and 1, respectively. Although their data followed the same general trends as that of other authors, the optical properties were only measured up to 900 nm at which point absorbance by all skin types was similar. However, the optical properties of FSTs I and V were not described by these authors. Between 400 and 900 nm, absorbance for FST VI was greater than the remaining skin types. FSTs II and III had the least absorption, with both spectra being similar and FST IV absorption was between that of the palest and darkest skin. Scattering for FST II was greatest and least for skin type VI up to 800 nm; beyond this all skin types had similar spectra. Absorption by the darkest skin was similar to that measured by Bosschaart et al.,^42^ in the 450 to 600 nm spectrum and similar to measurements made by Tseng et al.,^41^ beyond 850 nm. However, unlike Tseng et al.,^41^ Saager et al.^39^ measured a sharp decrease in absorption between 600 and 850 nm. Scattering measurements by Tseng et al.^41^ for FST V–VI were greater than those measured by Saager et al.^39^ by twofold at 600 nm down to 18% at 900 nm.

The reduced scattering and absorption coefficients were determined between 471 and 851 nm by Phan et al.^49^ for 15 subjects of all FSTs at varying locations. These data were not plotted in Fig. 4 because the peaks usually associated with the skin chromophores were not observed; however, the measurements were taken from one subject from FST I, three from FST II, six from FST III, three from FST IV, and two from FST V–VI. Absorption tended to decrease with increasing wavelength in all skin types. In general, subjects with skin types I and II had the least absorption in the visible spectrum. Subjects with skin types IV, V, and VI had the greatest absorption in the visible spectrum although there was some overlap with the data for skin type III.

The published data plotted in Fig. 4 suggest that absorption is generally greater for FST III–IV than I–II in the 400 to 450 nm spectrum, and in this region absorption by FST V–VI is less than for either of the lighter skin type groups (i.e. FST I-II and III-IV). It seems unlikely that absorption by FST V–VI would be less than by lighter FSTs due to the expected absorption by melanin and therefore differences may be attributed to author methods, sample numbers and locations, and potentially to determination of FST being subjective. Among the published data, only three authors produced data associated with FST V–VI compared with 15 datasets associated with the paler FSTs; this may be due to the prevalence of skin cancers in individuals of this skin type making it a research focus, or the country in which the research is taking place. Some studies with large sample numbers have been associated with the paler FSTs, for instance, the maximum sample number for FST V–VI is 6.^41^^,^^42^^,^^44^^,^^46^^,^^48^ For these reasons, we question the applicability of these data to skin types containing more melanin in their epidermis, i.e., FST V plus. Finally, not all useful optical properties have been measured at a wide range of wavelengths for all skin types in similar locations and there is variability in the wavelength ranges used by different authors and the measurements taken.

### 3.2 Variability Among Absorption and Scattering Coefficients: Published Values Combined

**Fig. 5:**
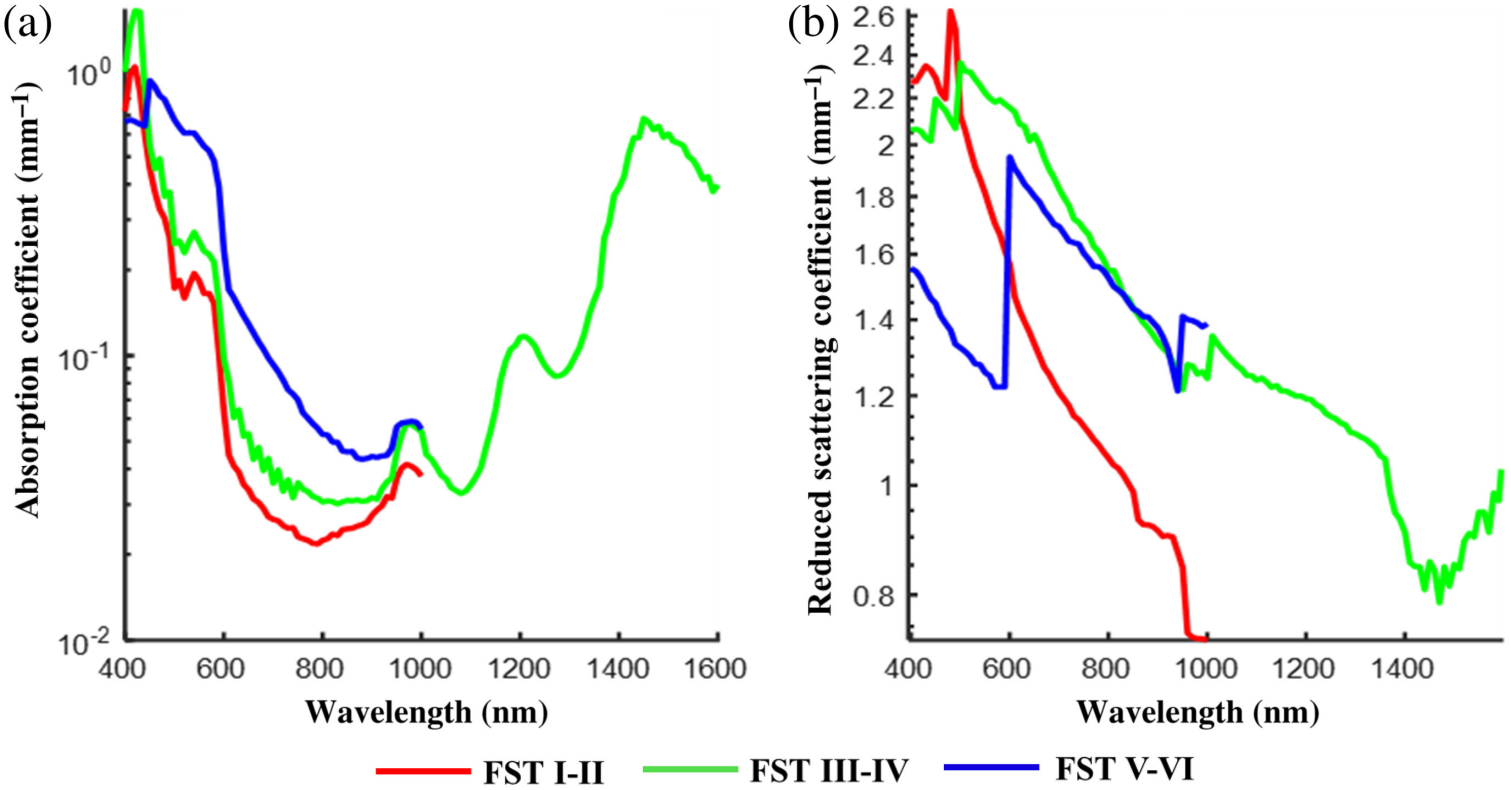
Differences between averaged published (a) absorption and (b) reduced scattering coefficients for FST I–II,^37^^–^^43^^,^^48^ III–IV,^39^^,^^41^^,^^42^^,^^44^^–^^47^ and V–VI.^39^^,^^41^^,^^42^

Figure 5 shows a comparison between absorption and reduced scattering coefficients for averaged total published data from FST I–II, III–IV, and V–VI. Data from one publication by Svaasand et al.^40^ were not included in the average measurements for the reasons discussed by Lister et al.^50^ Briefly, reduced scattering and absorbance measurements were only taken for FST I–II and are considerably higher than those measured by other authors being ∼80% greater than the average absorption measured by other authors and ∼97% greater than the average reduced scattering across the measurement spectrum, as can be seen in Fig. 4. Therefore, although the data included are very variable, this dataset is an extreme outlier and would potentially unrealistically skew the average data and hide differences that might be possible to determine between FSTs. FST V–VI absorption coefficients are ∼7% to 74% greater than those for FST I–II in the 400 to 1000 nm spectrum, the difference being least at 440 nm (6.5%) and greatest at 640 nm (74.4%). In the NIR absorption, coefficients start to converge but tend to increase in value with increasing wavelength. However, data are limited in this region, and beyond 1000 nm measurements were only available for FST III–IV. Therefore, it would be useful to gather further data for wavelengths beyond 1000 nm to determine whether the optical coefficients for the varying FSTs continue to converge with increasing wavelength. Beyond 1000 nm, data published for FST III–IV show absorption peaks associated with water.

Reduced scattering coefficients decrease with increasing wavelength for all FSTs. The data suggest that there are fewer scattering events in more melanized skin types at visible wavelengths (400 to 600 nm), where the reduced scattering coefficients for FST V–VI were up to twofold less than for FST I–II. The observed scattering differences in the visible spectrum are most likely to be due to absorption differences among skin types in this spectrum affecting scattered photon number and detection, i.e., FST V–VI absorbs more light in the visible region, therefore, proportionally fewer photons are available to be scattered and detected than in FST I–II. However, it is difficult to draw any conclusions from this due to the large variability in the published data contributing to this. It should also be noted that the data contributing to this region of the graph come from a single publication and sampled from only one subject leading to a marked skew in the data. Beyond 600 nm, there was a tendency toward convergence of the reduced scattering coefficients for FSTs III–IV and V–VI and between 600 to 1000 nm the reduced scattering coefficients associated with FST I–II were 22% to 48% less than for the darker FSTs. Reduced scattering coefficients were most similar between the palest and darkest FSTs at 600 nm, where the difference was 20%.

### 3.3 Transport Mean Free Path of Light Through the Skin

**Fig. 6:**
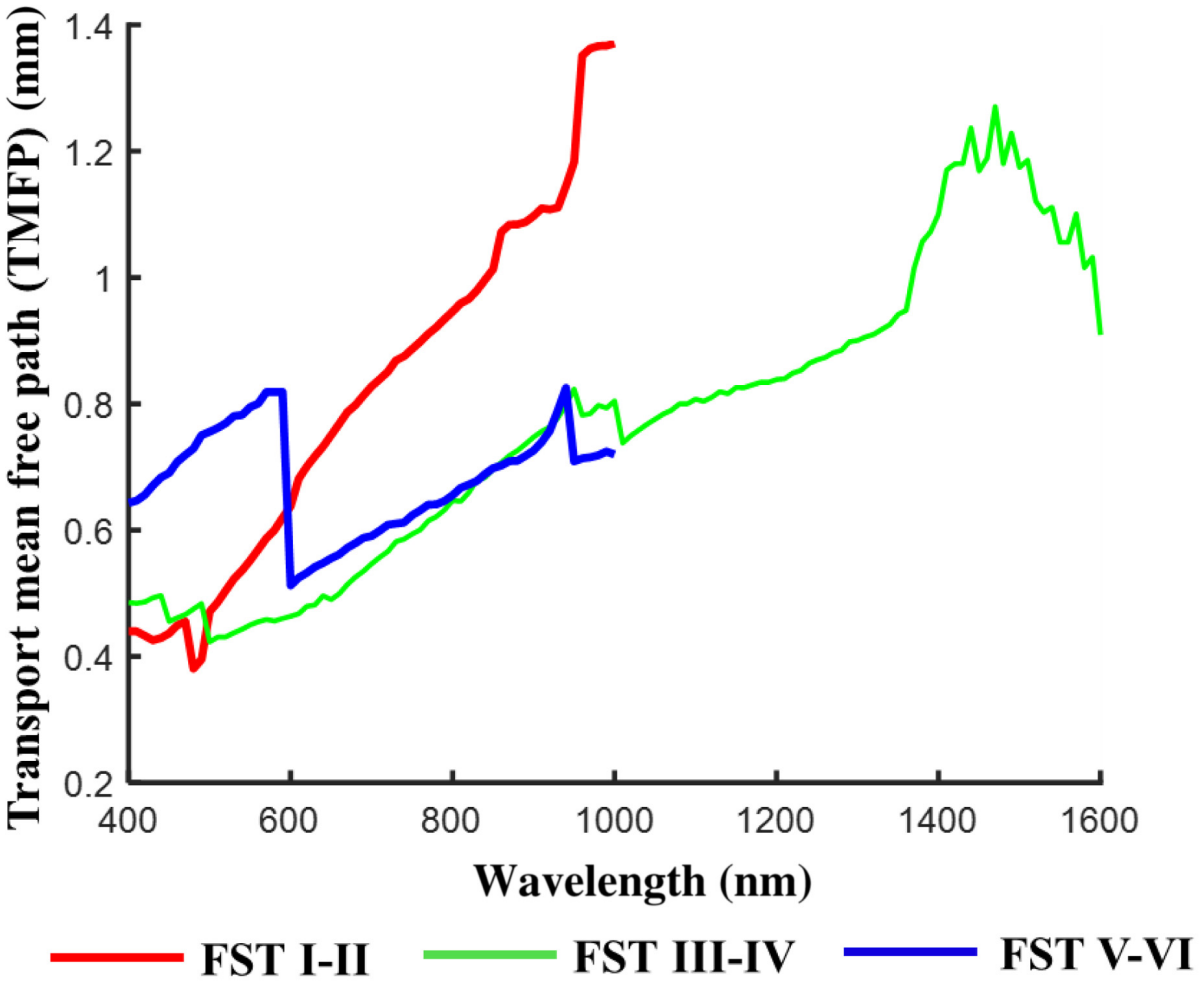
Average TMFP (mm) associated with each FST group.

To put the values presented in Sec. 3.2 into context, we consider the maximum depth at which it would be possible to focus light into skin for different types and hence how deep into skin an image can easily be formed (see Fig. 6). The transport mean free path (TMFP) was used to provide a measure of this depth, where $\mathrm{TMFP}=1/\mu_{s}^{\prime }$ and $\mu_{s}^{\prime }$ is the reduced scattering coefficient given by $\mu_{s}^{\prime }=\mu_{s}(1-g)$, where $\mu_{s}$ is the scattering coefficient of the material and g is the anisotropy factor representing the average cosine of the scattering angle.^51^ The TMFP predicts how far light will travel through a material before it becomes diffuse with the direction of propagation no longer resembling the starting direction of the beam. At this depth, it is virtually impossible to focus the light with standard optics and without the use of wave-front correction or adaptive optics.

The results gathered from the published data and analyzed here suggest that beyond 600 nm the depth at which an image could potentially be formed increases with increasing wavelength and is greatest for FST I–II at 980 to 1000 nm being 1.37 mm, for FST III–IV at 1470 nm being 1.27 mm, and for FST V–VI at 940 nm being 0.83 mm. For comparison, the maximum TMFP for FST III–IV in the 400 to 1000 nm range is 0.82 mm at 950 nm. Because the TMFP is related to scattering, and the NIR is the region where scattering is least (Sec. 3.2), this is the region where light is likely to travel the furthest distance into the skin. Like scattering, differences between the TMFP for FST III–IV and V–VI are least in the NIR spectrum. The observed TMFP magnitude in the 400 to 600 nm spectrum is unlikely to be realistic as described in Sec. 3.2. There is the biggest difference between FST V–VI and FST I–II between 960 to 1000 nm where the TMFP is ∼90% greater for FST I–II than the darker FSTs. Accounting for the above, in the 400 to 1000 nm spectrum, it is likely that light of between 940 and 1000 nm may provide a useful wavelength for imaging skin of different FSTs at depth. However, there is a small peak in absorption at these wavelengths as shown in Fig. 5, and because TMFP calculations do not take absorption into consideration it is likely that the amount of light reaching these depths will be affected. Absorption coefficients are, however, at least an order of magnitude less than reduced scattering coefficients at these wavelengths so absorption may have little effect on the distance light can travel through the skin or the amount of light that can reach the depths. This is discussed further in Sec. 3.4

We are unable to determine whether the TMFP may increase further beyond 1000 nm as shown for FST III–IV because comparative data for FST I–II and V–VI do not exist at these wavelengths among the published data used for this review. It would, therefore, be useful to gather data beyond a wavelength of 1000 nm for all FSTs to determine if the optical coefficients decrease with increasing wavelength and to therefore determine if the maximum imaging depth can be extended for longer wavelengths.

The average TMFP across each FST of photons travelling through the skin ranges from 0.68 to 0.81 mm across the measurement spectrum depending upon FST. Using the published data, the average TMFP is least in FST V–VI samples and greatest in FST III–IV in the 400 to 1000 nm spectrum. It should be noted that TMFP does not consider absorption and a beam travelling through FST V–VI skin would likely be more affected by attenuation due to absorption than FST I–II. In practice, the power of the light source could be increased to account for absorption losses but a consequence of this would be an increase in the risk of photodamage to the tissue.

### 3.4 Transmission of Light Through the Skin

Estimating how much light will reach a certain depth into the skin is important for medical treatments that use light and photons such as PDT. With scattering present it is accepted that beyond the mean free path a form of the Beer Lambert law can be written with the attenuation written as $e^{-\mu_{s}^{\prime }l}$, where depth is l and the reduced scattering coefficient is $\mu_{s}^{\prime }$.^52^^,^^53^ When absorption and multiple scattering is present, the situation is more complicated because the total propagation path of the detected photons is generally larger than l (the penetration depth). Monte Carlo simulation can be used to simulate the path length of the photons arriving at a particular detection position, which can be recorded. In practice, the additional propagation length is a function of the scattering coefficient, the penetration depth, and the detection mechanism. This is discussed by Sassaroli and Fantini,^54^ and elsewhere.

For our purposes, to come up with some plausible values, we consider the case where we wish to focus the optical beam to a well-defined position as is the case with, for example, PDT or nonlinear microscopies (such as two photon harmonic, etc.). In this case, it is reasonable to say that the light available at a certain depth to form a focus has suffered far less scattering than a “typical” photon. Bearing this in mind, the intensity of light reaching a depth, l, into a medium with a reduced scattering coefficient of $\mu_{s}^{\prime }$ and an absorption coefficient of $\mu_{a}$ can be expressed as $I_{\text{out}}=I_{\text{in}}e^{-(\mu_{a}+\mu_{s}^{\prime })l}$, where $I_{\text{in}}$ is the input intensity and $I_{\text{out}}$ is the intensity at a depth of l. In practice, this is represented as the percentage of light that has been transmitted or has reached l. We acknowledge that this is a somewhat ad hoc expression and will tend to overestimate the amount of light reaching a particular position, it will, however, give a useful ballpark and a method for considering the challenges of certain technologies with skin color.

**Fig. 7:**
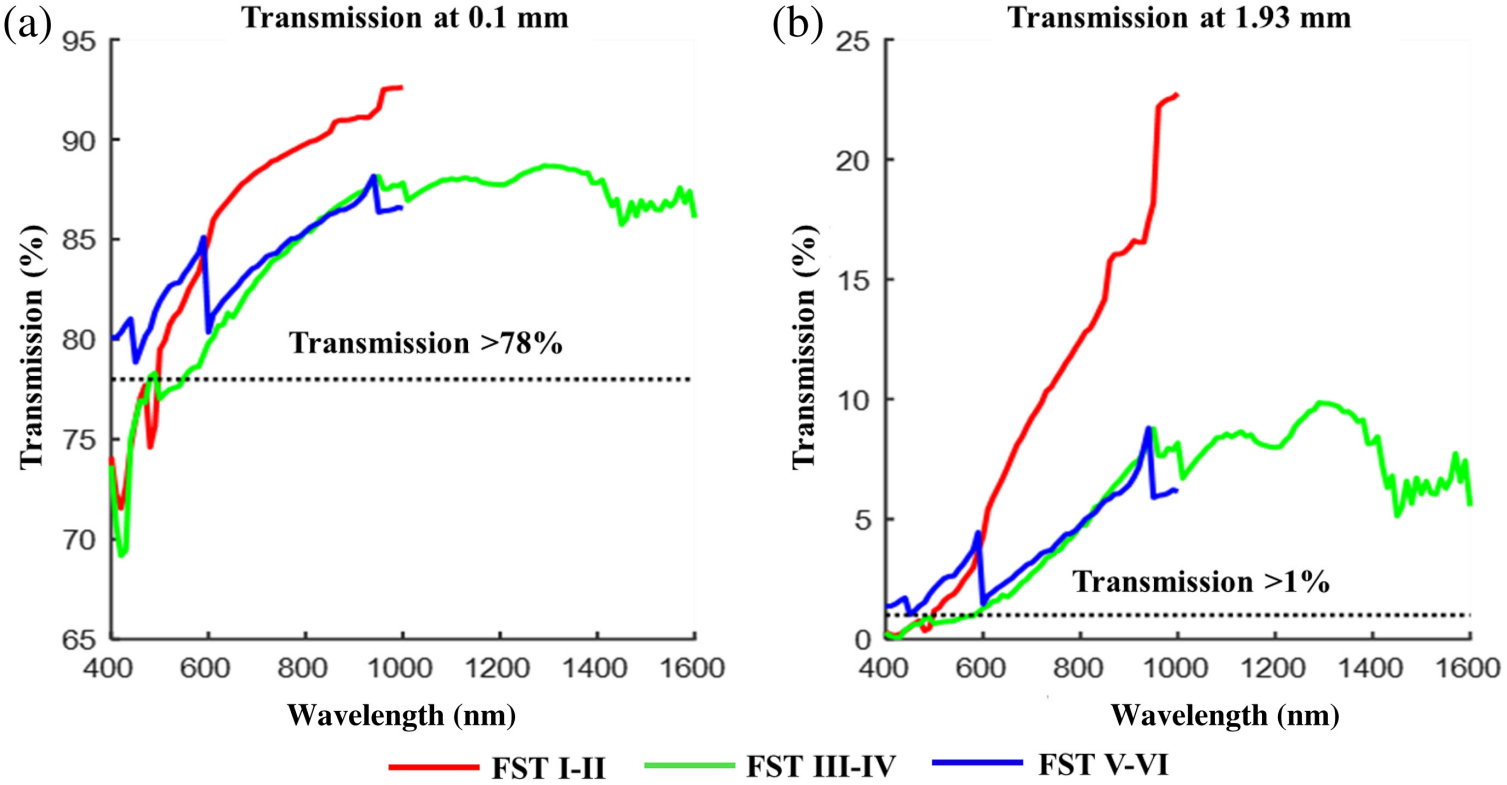
(a) Transmission of light at 0.1 mm* dependent upon skin type. (b) Transmission of light beyond 1.93 mm* dependent upon skin type. FST I–II, red; FST III–IV, green; FST V–VI, blue. *0.1 and 1.83 mm correspond to the average epidermal and dermal thickness respectively giving a total depth of 1.93 mm.^43^

The percentage of light propagating through given depths using averaged published data for each FST was calculated. Figure 7(a) shows that at all wavelengths measured in the published data, more than 69% of light was transmitted beyond a penetration depth (l) of 0.1 mm, which corresponds to the average thickness of the epidermis.^43^ However, the percentage of light transmitted beyond this depth was variable both for wavelength and skin type. In general, between 600 and 1000 nm light travels further into FST I–II skin than FST III–IV and V–VI skin according to TMFP calculations as shown in Fig. 6. Similarly, the percentage of light transmission was shown to be greatest for FST I–II in this spectral region. The maximum amount of light transmitted beyond 0.1 mm is 92.6% at 1000 nm, 88.7% at 1290 nm, and 88.2% at 940 nm for FST I–II, III–IV, and V–VI, respectively. The measured spectral range for FST III–IV skin is much broader than for the other two skin type groups; if the same range is considered (400 to 1000 nm) then the maximum light transmitted through this group is 88.2% at 950 nm. The percentage transmission for FST III–IV and FST V–VI is very similar to each other in the 600 to 940 nm wavelength range. The data contributing to the transmission calculations are extremely varied as shown in Fig 4; averaging of these is likely to have contributed to the similarity of the reduced scattering coefficients and therefore the percentage transmission, which is largely affected by transmission, in the 600 to 940 nm range.

It might be expected that light transmission through all FSTs would be maximal at 1000 nm (and potentially even longer wavelengths if the data were available). However, due to the variability and limited nature of the data that make up the averaged coefficients used for this work, it is difficult to reliably and precisely extract information. Calculations suggest that 940 to 1000 nm may be a useful wavelength range for enabling most light to be transmitted through the skin as also suggested in Sec. 3.3. Despite the increasing absorption observed beyond 940 nm (described in Sec. 3.2), because scattering is least for all FSTs at these wavelengths, transmission is greatest in this spectrum. Although this region coincides with water absorption peaks, because absorption has much less of an effect than scattering, absorption by water may not have a significant negative effect on transmission.

Based on the published data, largest losses of light occur with increasing skin depth; across the 400 to 1000 nm spectrum on average ∼14% of light is lost at a depth of 0.1 mm in FST I–II and by a depth of 1.93 mm 91% of the remaining light has been lost. In FST III–IV and V–VI skin, these losses are on average 18% and 16%, respectively, at 0.1 mm and 97% and 96%, respectively, by a depth of 1.93 mm. For FST III–IV beyond 1000 nm less light is lost than in the 400 to 1000 nm spectrum, with 12% average losses at a depth of 0.1 mm and 92% by a depth of 1.93 mm. Because data are not available for FST I–II and V–VI beyond 1000 nm, we are unable to determine whether less light is lost with increasing wavelength as shown for FST III–IV. To put these losses into context, if a biomedical technology requires 10 mW of light to be transmitted beyond a depth corresponding to the average combined depth of the epidermis and dermis (1.93 mm), the skin must be illuminated with ∼406.5  mW of light (this is based on FST III–IV, where average light losses are greater than FST I–II and V–VI in the 400 to 1000 nm spectrum). However, losses are wavelength dependent as well as FST dependent and since most light is lost at shorter wavelengths the power used to illuminate the skin would need to be much greater at these wavelengths (up to 18 W). By comparison, beyond 940 nm the power required to illuminate the skin and have 10 mW remaining beyond the epidermis and dermis is as low as 47 mW for FST I–II. However, despite being least at these wavelengths, more than twice this power would be required for FSTs III–IV and V–VI.

More than 1% of light was transmitted beyond the full depth of the skin (1.93 mm) at all wavelengths measured for FST V–VI. However, in the 600 to 900 nm spectrum, the percentage of light transmitted beyond this depth was greater for FST I–II than for FST V–VI. This coincides with the region discussed in Sec. 3.1 where elevated scattering by FST V–VI in the 600 to 800 nm region is attributed to the scattering power of melanosomes.^41^ For more than 1% of light to be transmitted beyond the skin, at least 78% of light must be transmitted beyond 0.1 mm [Fig. 7(b)]. For FST I–II and III–IV, this is most likely to happen beyond 600 nm. More than 78% of light was transmitted beyond 0.1 mm for FST V–VI at all wavelengths measured [Fig. 7(a)] and therefore, more than 1% of light has the potential to be transmitted beyond skin with a total depth of 1.93 mm, the average combined depth of epidermis and dermis described by Meglinski and Matcher.^43^

Light transmission beyond 0.1 mm was the least at the shortest wavelengths and the minimal transmission reported for each FST in the visible wavelength range being 78.9% for FST V–VI (at 450 nm) compared with 71.2% for FST I–II (at 420 nm) and 69.2% for FST III–IV (at 420 nm). In the 400 to 1000 nm spectrum, transmission was greatest through all FSTs at wavelengths longer than 940 nm. At 940 nm, light is still travelling predominantly in the forward direction (as shown by TMFP calculations) at a depth of 0.1 mm and is at ∼88% on average of its original intensity for all FSTs. At this wavelength and a skin depth of 1.93 mm, light transmission has reduced to between 18% and 23% in FST I–II compared with between 8% and 9%, and between 6% and 9% in FST III–IV and V–VI, respectively.

Nishimura et al.^55^ described how the penetration depth of optical measurements changes significantly at wavelengths corresponding with strong water absorption at longer wavelengths. They found that the penetration depth is less than 1 mm in the 1400 nm water absorption band, which means that at this wavelength light would not penetrate beyond the skin.

By modeling photon transmission and using data taken from published results for the optical properties of the skin, Finlayson et al.^56^ showed that 1% of light reached depths of 1.6 and 5 mm at wavelengths of 450 and 650 nm, respectively. Similarly, using Monte Carlo (MC) modeling, Ash et al.^57^ showed that at wavelengths of 450 and 650 nm 1% of light has the potential to reach 1.6 and 4.75 mm, respectively.

Sordillo et al.^58^ described the potential of longer wavelength light to follow a linear trajectory for a greater distance than shorter wavelength light, due to a reduction in scattering and absorption coefficients. For published data assembled by Shaw et al.,^59^ it was shown that light in the 1000 to 2000 nm spectrum can increase penetration depth into biological tissues, improve image resolution, reduce tissue autofluorescence, and aid image-based diagnostics. For this reason, gaining a set of data relating to the optical properties of the skin beyond 1000 nm for different FSTs would be beneficial.

The results of analysis of the published data described in detail in this section are summarized in Table 2. Note that the wavelengths shown are those that are important for the optical medical technologies that are described in Sec. 4.

**Table 2:** Summary of the average absorption and reduced scattering coefficients (${\mathrm{mm}}^{-1}$), calculated TMFP (mm) and percentage transmission at depths corresponding to the average epidermis (0.1 mm) and the combined epidermis and dermis (1.93 mm). Data are given at wavelengths important to OCT, PDT, and medical wearables.

Coefficient	FST	Wavelength (nm)						
		410	620	660	780	940	1000	1300
Average $\mu_{a}$ (${\mathrm{mm}}^{-1}$)	FST I–II	0.975	0.041	0.031	0.022	0.032	0.038	—
	FST III–IV	1.396	0.061	0.043	0.032	0.037	0.054	0.091
	FST V–VI	0.675	0.160	0.120	0.058	0.047	0.055	—
Average $\mu_{s}^{\prime }$ (${\mathrm{mm}}^{-1}$)	FST I–II	2.275	1.427	1.301	1.085	0.873	0.729	—
	FST III–IV	2.065	2.086	2.003	1.609	1.253	1.243	1.111
	FST V–VI	1.546	1.878	1.781	1.561	1.212	1.389	—
TMFP (mm)	FST I–II	0.44	0.7	0.77	0.92	1.14	1.37	—
	FST III–IV	0.48	0.48	0.5	0.62	0.8	0.8	0.9
	FST V–VI	0.65	0.53	0.56	0.64	0.83	0.72	—
% Transmission at 0.1 mm	FST I–II	72.55	86.35	87.52	89.52	91.35	92.62	—
	FST III–IV	70.74	80.68	81.5	84.87	87.9	87.84	88.67
	FST V–VI	80.08	81.56	82.69	85.06	88.17	86.55	—
% Transmission at 1.93 mm	FST I–II	0.19	5.88	7.64	11.8	17.44	22.75	—
	FST III–IV	0.13	1.59	1.93	4.21	8.29	8.18	9.83
	FST V–VI	1.38	1.96	2.55	4.4	8.81	6.16	—

## 4 Importance of Accurately Determining the Optical Properties of Skin of Different FSTs: Three Case Studies

**Fig. 8:**
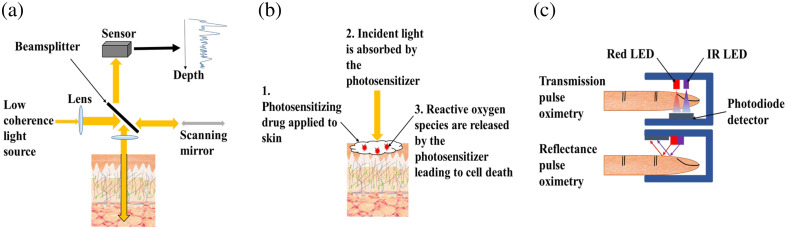
Three commonly used optical medical technologies: (a) time domain OCT, (b) PDT, and (c) PO as an example medical wearable.

Due to the differences discussed in Sec. 3 between the optical properties of dark versus light skin and the potential effect these have on penetration depth and transmission, we will investigate the effect of FST on three commonly used optical medical technologies, OCT, PDT, and medical wearables with a focus on PO, in Secs. 4.1–4.3. The schematics in Fig. 8 provide a summary of how these technologies work.

The key effect of FST on these technologies is highlighted in Fig. 3. Table 3 shows the typical wavelengths at which the optical techniques discussed in this section are used in clinical situations.

**Table 3:** Clinical uses of optical imaging of the skin and optical therapies in healthcare and the wavelengths at which they are used.

Clinical use	Method	Wavelength used	Affected by skin color	Ref.
Dermatological imaging	OCT	1310 nm	Image quality may be reduced in patients with darker skin	8
Treatment of AK	OCT	1300 nm	Not discussed	60
Treatment of AK	ALA-PDT	410 to 420 nm	Not discussed	61
Treatment of AK	PDT	Broadband daylight	Not discussed	62
Treatment of BCC	MAL-PDT	Broadband (560 to 740 nm)	Not discussed	63
Treatment of BCC	ALA-PDT	410 to 420 nm	Not discussed	61
Treatment of SCC	MAL-PDT	630 nm	Not discussed	61
Treatment of cutaneous T cell lymphoma—mycosis fungoides	MAL-PDT	630 nm	Not discussed	64
Treatment of acne	MAL-PDT	630 nm	Regimen optimization required for different FSTs	65
Treatment of BCC	Chlorin	662 nm	Not discussed	66
Treatment of melanoma	Chlorin	662 nm	Not discussed	67
Measurement of oxygen saturation	PO	622 to 780 nm and 780 to 2400 nm	Darker skin tones are more likely to have overestimated oxygen saturation levels	10
Heart rate sensing	PPG	542 nm	Not discussed	68

In addition to the three optical medical technologies discussed in Secs. 4.1–4.3, there are multiple other optical technologies, both older and emerging, including photoacoustic, multispectral, and dermoscopy to name a few. Dermoscopy is a noninvasive method for visualizing skin lesions due to skin cancers and it consists of a hand-held instrument with an integrated light source and a 10× magnification lens, which enables visualization of the epidermis and superficial layers of the dermis.^69^ Dermoscopy is affected by FST and is optimal for FST I–II.^70^ It is currently used by dermatologists but not recommended for use by GPs due to specialist training requirements.^71^ Photoacoustic imaging involves the absorption of the illuminating optical beam to generate acoustic signals (which are less affected by scattering than photons) enabling deeper imaging than using light alone; however, resolution is limited when compared with optical imaging.^72^ Photoacoustic imaging allows the distinction of skin layers and, in combination with OCT, imaging depths of 5 mm have been observed; however, depth penetration is affected by skin color.^9^^,^^72^^,^^73^ Multispectral imaging builds up spectral images of a tissue from reflected light at multiple wavelengths in the 400 to 955 nm range and provides information regarding the concentration of the skin chromophores at a given site.^74^ This has shown potential for assessing skin cancers by providing information regarding the lesion properties, such as molecular composition preventing the need for invasive biopsy assessment, and surgical tumor removal in conjunction with fluorescent markers.^75^^,^^76^ However, the high proportion of nonmalignant lesions being classified as melanoma has caused this technique not to be used clinically.^77^ This technique is affected by skin color; however, collecting the spectral properties at longer wavelengths may help to overcome absorption problems associated with skin color.^78^^,^^79^ The advantages and disadvantages of some of these technologies are discussed further by Kratkiewicz et al.^80^

### 4.1 Optical Imaging Using OCT

Optical imaging is an important technique because it significantly reduces a patient’s exposure to ionizing radiation from, for example X-rays, and can therefore be used repeatedly for monitoring disease progression and treatment results.^81^ There are multiple forms of optical imaging and a recent review compared OCT, reflectance confocal microscopy, and multiphoton microscopy as techniques for noninvasive optical biopsy of *in vivo* skin of patients with pigmented lesions, such as melanoma and basal cell carcinoma (BCC).^82^ These all use wavelengths beyond 700 nm and therefore avoid maximal melanin absorption reducing the effect of skin color. Of these, OCT provides the greatest imaging depth and field of view.

OCT produces three-dimensional images noninvasively using low coherence interferometry to image within optically scattering samples, such as biological tissues. In OCT, the beam is split in two to give a reference beam, normally reflected off a mirror, and a sample beam, backscattered from the tissue of interest, and the interference between the two beams is measured. The use of interferometry means that photons that have been scattered multiple times and are incoherent are rejected, and so will not obscure the image by contributing to background noise. OCT is sensitive to scattering from tissues because coherence is essential for detection; OCT detects back-reflected light that is coherence matched with the incident light.^83^^,^^84^ OCT imaging at depth is negatively affected by absorption with light absorption and attenuation in upper tissue layers leading to less light reaching lower layers. Light reaching these lower layers can also become attenuated on its return prior to detection.^85^ Widefield imaging methods can only image samples to depths of ∼20  μm; however, OCT can be used to image to depths of up to 2 mm with resolution smaller than 10  μm.^83^^,^^86^ OCT is currently unable to image much deeper than a few millimeters because beyond this depth the proportion of light that has not under gone multiple scattering events is too small to be detected.

OCT has been adopted most commonly for obtaining noninvasive high-resolution images to monitor eye health (e.g., macular degeneration, retinopathy, and optic nerve neuropathies). However, other clinical applications include obtaining detailed images of coronary arteries, guiding treatment, and monitoring stents in cardiovascular diseases; OCT in combination with endoscopy to detect and guide treatment of pancreatic cancer; as well as becoming more commonly used in dermatology over recent years including diagnosis and monitoring of skin disorders.^86^^–^^95^

Because OCT resolution and imaging depth are affected by absorption, the melanin content of the skin and its effect on absorption need to be considered. In the field of dermatology, OCT is most extensively used in the diagnosis of BCCs; however, it currently does not have the accuracy to diagnose malignant melanomas.^96^ It has also been shown to have potential for monitoring therapy for skin disorders such as scleroderma, psoriasis, eczema, and wound healing.^96^^–^^98^ The skin layers from the stratum corneum to the upper dermis, and skin appendages and blood vessels can be clearly imaged.^95^ Although OCT has been shown to accurately image the development of actinic keratosis (AK), a precursor to squamous cell carcinoma (SCC), in patients of FST I–III, the majority of studies do not mention skin color and this is likely due to the perception that AK only affects people with light skin.^99^^–^^104^ However, although AK is more common in people with light-colored skin, it can be found in patients with all FSTs.^105^

The effect of FST on OCT imaging has been rarely investigated. A 2019 publication suggested that image quality may become slightly lower with increasing pigmentation, but concluded that the difference in imaging quality and depth was not significant among all FST.^8^ Here, OCT with a center wavelength of 1310 nm was used and a depth of 1.3 mm below the skin surface was imaged. The authors suggest that despite having quantitatively lower quality, where light intensity at this depth was significantly less in dark skin than light skin, imaging is qualitatively indiscriminate for skin color. However, although melanin absorption should be minimal in this region, as described in Sec. 2, melanin may still have absorbing properties even at this wavelength.^31^ Ekelem et al.^8^ suggested that further work is required to study the effect of skin type on OCT imaging, and that their small sample size [16 subjects in total; FST I–III (n=8) and FST V–VI (n=8)] and sun-exposure of the subjects may have influenced results.

The effect of skin color on OCT has rarely been discussed making it difficult to draw conclusions. This is presumably because OCT uses longer wavelengths, which are supposed to be unaffected by absorption by melanin. However, the majority of studies have also been in patients with FST I–II; although OCT tends to use wavelengths beyond 1300 nm for skin imaging, darker skin has rarely been studied and it has been suggested that OCT image quality may be reduced in patients with darker skin but further investigation is required.^8^

### 4.2 Photodynamic Therapy

PDT involves the use of light sensitive medications to destroy abnormal cells and can be used to treat some diseases of the skin such as actinic keratoses and BCC, warts and acne as well as macular degeneration of the eye, and cancers of the esophagus, mouth, and lung.^62^ For PDT to be effective, a clear understanding of the propagation of light through human tissue is required, which is affected by local absorption and scattering coefficients.^3^ In general, the scattering coefficients of tissues decrease monotonically with increasing wavelength; however, absorption coefficients vary greatly depending upon the absorbing chromophores present. For example, hemoglobin in the blood and melanin in the skin are strong absorbers of visible light, whereas water has multiple absorption peaks at infrared wavelengths. PDT can be applied either superficially to noninvasively treat accessible regions such as the skin; invasively, where superficial applications would have limited light penetration, to treat bulk tissues with optical fibers being placed directly in the tissue (interstitial treatment); or within a cavity such as the bladder via an endoscope.^3^

PDT requires that a photosensitive drug is activated in the presence of light and oxygen to produce reactive oxygen intermediates that irreversibly damage cells.^61^ This allows selective treatment; however, selectivity is affected by uptake of the photosensitive drug by target cells and its metabolism to an active form as well as by penetration of the light source. This is, in turn, affected by the optical properties of the tissue and, for treatment of a skin-disorder, the optical properties are affected by FST. Inflammatory hyperpigmentation is a side effect more commonly associated with the treatment of patients with FST IV–V and caused by excess synthesis and deposition of melanin enhanced by inflammatory responses.^20^ Potential reasons for the common association of inflammatory hyperpigmentation with dark skin are increased oxidative stress and reduced vasodilation, which are improved with vitamin D (the intrinsic production of which is reduced in darker skin), as well as increased circulating inflammatory markers, which could lead to impaired melanin production and altered skin cell activity.^106^

A wide variety of wavelengths are used for PDT. Blue light (450 to 495 nm) is used to maximize absorbance and longer wavelengths of light (beyond 620 nm) have best tissue penetration.^107^^,^^108^ However, photosensitive drugs are only able to generate singlet oxygen, the most destructive reactive oxygen species, in the presence of light from 600 to 800 nm.^109^

Two photosensitizing drugs are commonly used for PDT and the treatment of skin conditions; aminolevulinic acid (ALA) and its methyl ester cream [methyl aminolevulinate (MAL)]. Both are absorbed into the skin becoming converted to protoporphyrin IX with a major absorption peak in the blue light spectrum (410 to 420 nm).^61^^,^^110^ However, due to penetration, red light (635 nm) is usually used to activate protoporphyrin IX for skin cancer treatments.^111^ Because MAL is more lipophilic than ALA it is absorbed more deeply into the skin and is therefore commonly used in conjunction with red light, which penetrates more deeply into the skin than blue light.^112^ Treatment with MAL-PDT has most commonly been associated with treatment of nonmelanoma skin cancers and, more recently, acne.^112^^,^^113^ A further photosensitizing drug, chlorin, comprising of chlorin e6, chlorin p6, and purpurin 5, has been approved for use in Russia and Republic of Korea with absorption at 662 nm and has been shown to be less painful to patients than MAL and ALA treatments.^66^^,^^67^^,^^114^ Because light penetration is limited in the visible range, these drugs are used for the treatment of superficial skin diseases with therapeutic depths of <2  mm; AK, for example, is usually <300  μm deep.^115^^,^^116^

Patients with FST IV–VI are at a greater risk of epidermal injury and hyperpigmentation or hypopigmentation due to PDT than those with light skin due to laser energy absorption by melanin at the wavelengths used for treatment.^20^ Accurate determination of the individual’s skin properties and the associated absorption and scattering coefficients is required to reduce adverse effects by personalizing treatment regimens including reducing the doses of light, the strength of the photosensitive drug, and the incubation time of the drug on the skin.^65^^,^^117^

For the treatment of acne, the total dose of red light needs to be adjusted for FST as discussed by multiple authors for patients of Asian origin.^65^^,^^118^^–^^120^ However, there has been very little research into patients with the darkest skin types.^121^ Where PDT has been used to treat nonmelanoma skin cancers, including BCC, SCC, Bowen’s disease, and AK, the majority of the published work has studied efficacy in people with FST I–II.^112^^,^^122^^–^^125^ This is probably because skin cancers are more prevalent in pale-skinned populations with the people with the darkest skin being diagnosed, with approximately one twentieth of the cases of skin cancer of those with the palest skin.^126^ Nevertheless, there are incorrect perceptions that skin cancers are unique to the lighter-skinned population and morbidity in darker-skinned people is greater due to later diagnosis, with 70% 5-year survival rate in dark-skinned people compared with 92% in lighter-skinned people.^127^ However, incidence and morbidity are difficult to determine accurately due to limited availability of data in people with dark skin.^128^ Farberg et al.^129^ suggested that to evaluate the efficacy of PDT accurately, clinical studies should represent a greater diversity of FSTs, age groups, and anatomic locations.

PDT has mostly been investigated in light skinned patients and this is probably due to its use in skin cancer treatment and the increased likelihood of skin cancer in these patients.^124^

### 4.3 Medical Wearables and PO

Medical wearable devices are worn directly on the body to measure vital signs including heart rate, blood pressure, and oxygen levels. In healthcare, the most widely used method for optical sensing is photoplethysmography (PPG), which monitors blood flow in real time.^130^ Differences in the interaction of light with human tissues at differing wavelengths is exploited to provide information including oxygen saturation, blood pressure, and more recently other cardiovascular-related diseases.^131^ The PPG signal can either be measured in transmission, where the light emission is physically opposite to the detection apparatus, or reflection, where the light emission and detection apparatus are on the same side as each other. In transmission, the signal from high capillary regions such as earlobes and fingers is most commonly measured. Light of 680 or 810 nm is used due to its penetration depth and ability to differentiate between oxygenated and deoxygenated blood. The light intensity transmitted through the skin will depend upon the volume of blood at the measurement site. For reflectance measurements used, for example, for heart rate monitoring, green light is usually used as this provides high quality PPG signals and is less affected by temperature changes and accuracy.^132^

Currently, the most common form of PPG is PO, which works in transmission and uses two light sources to measure oxygen saturation of the blood.^10^ At the wavelengths used for PO, our calculations suggest that the TMFP for FSTs I–II, III–IV, and V–VI are 0.92, 0.61, and 0.64 mm, respectively. Oxygen saturation is estimated as a ratio of ratios of the minimum (DC: non-pulsatile ‘direct current’ blood component) versus peak (AC: pulsating ‘alternating current’ arterial blood components) light emanating from oxygenated blood, which transmits red light (622 to 780 nm), and deoxygenated blood, which transmits infrared wavelengths (780 to 2400 nm) as follows

$$R={\left(\mathrm{AC}/\mathrm{DC}\right)}_{1}/{\left(\mathrm{AC}/\mathrm{DC}\right)}_{2}$$

where AC is the signal amplitude and DC is its baseline, and 1 and 2 correspond to the red and infrared measurements.^133^

A clear understanding of the optical properties of the tissues being measured is required to be able to interpret the results appropriately. A recent flurry in publications identifies that skin pigmentation affects the accuracy of PO.^11^^,^^134^^–^^140^ However, issues were identified in 2007 by Feiner et al.,^141^ who suggested that PO overestimates oxygen saturation in darker skin tones and up to 10% differences in saturation estimates were observed among different instruments. Using the ratio of ratios of pulsatile light to total transmitted light at both red and infrared wavelengths oxygen saturation of arterial blood is estimated. In theory, because the measurements should only be dependent upon arterial saturation, the estimated oxygen saturation should be independent of FST and other variables.^141^ However, where oxygen saturation is out of the normal range, this does not seem to be the case and absorption by melanin in darker skin appears to affect outcomes. Feiner et al.^141^ showed that at low oxygen saturation, the instruments overestimated the saturation levels in intermediate and dark-skinned subjects. To obtain a stable result from back scattered light being detected in PO, not only the blood’s absorption needs to be considered but also that of the skin. The attenuation of incident light used in PO to measure oxygenated blood (light in the red spectrum) by melanin implies that PO may be less reliable in dark skin.

The results of Feiner et al.^141^ and the reason that skin type needs to be considered have been exemplified by the recent COVID-19 pandemic.^134^^,^^142^^,^^143^ Black and ethnic minority groups had poorest outcomes from COVID-19 infection and this was compounded by the overestimation of oxygen saturation.^11^^,^^134^ The situation where actual oxygen saturation was <88% in patients but their measured PO calculations were >92% occurred three times more in Black patients than white meaning that many Black patients were at increased risk of undetected hypoxemia.^135^^,^^136^ A recent study comparing arterial blood gas (invasive) with PO showed that Black patients were at much higher risk of having occult hypoxemia (an arterial oxygen blood saturation of <88%) due to overestimation of blood saturation by PO thereby limiting access to supplemental oxygen for Black patients.^137^

At the wavelengths used for PO, our calculations suggest that the TMFP for FSTs I–II, III–IV, and V–VI are 0.92, 0.61, and 0.64 mm, respectively. Because depth penetration of light is likely to be less in individuals with darker skin tones, this may also be a contributing factor to the bias associated with PO.

Other wearable healthcare products used to measure heart rate and arrhythmia, for example, have also been shown to be limited by racial bias.^144^ Colvonen et al.^144^ expressed concern that as optical wearable devices are becoming more common in healthcare, and as evidence mounts regarding reduced accuracy of these devices in darker skin, they may worsen healthcare disparities for the Black population. Colvonen et al.^144^ suggested that testing of optical wearable devices is often carried out in populations lacking diversity with inaccurate determinations of skin tone and that consumer wearable companies are aware of these flaws but doing little to address them. On the contrary, Bent et al.^145^ investigated the reliability of wearable optical heart rate monitors on 53 subjects with a variety of FSTs and showed that that these were unaffected by skin color. However, Colvonen et al.^146^ suggested that these results may have been due to small sample size and inaccuracies in Fitzpatrick skin color determination and are unlikely to reflect the true effect of skin color on the accuracy of optical wearable devices. Others also suggest that small sample sizes and inaccurate skin color determination may be leading to misinformation regarding the accuracy of medical wearables in people of color.^134^^,^^147^

## 5 Conclusions

The variation in optical properties with skin color is medically important, affecting optical medical technologies. An accurate measurement of skin color is essential for OCT imaging of, for example, skin cancers; for treatment of skin cancers and other skin conditions using PDT; and for medical monitoring using wearable devices. The skin is the first barrier to light for optical imaging, and imaging depth is dependent upon both its scattering and absorption properties, which depend on skin color and the wavelength of light.

Currently, published data do not reflect all skin colors equally. Data used in this review were collated from 12 publications, which have been cited between 5 and 673 times. Publications by Doornbos et al.^38^ and Meglinski and Matcher^43^ were the most cited with 673 and 544 citations, respectively. Both of these publications present optical coefficients for FST I–II skin. Publications describing the optical properties of only FST V–VI skin have been cited least in total, with an average of 121 citations per publication compared with an average of 296 citations per publication for FST I–II. It should be noted that the three publications representing FST V–VI used in this review also presented data relating to the lighter skin types and the total sample numbers for darker skin were less than for light skin among these publications. The publications used for this review that reported optical properties of light skin tones tended to be less recent, which might represent social changes and affect downstream uses of these properties.^148^

Better quantification of FST is required. Determination of FST is subjective; for the accuracy of future applications spectrometric determinations of skin type is required incorporating a direct measure of melanin content.

Scattering affects polarization of light and it should be noted that some medical optical imaging techniques use polarized light including polarization-sensitive OCT, polarized light elastic scattering spectroscopy, and cross-polarized light imaging.^149^^–^^151^ Polarization is affected by scattering, which alters the degree of polarization and, therefore, skin color has the potential to cause polarization changes due to scattering by different sized melanosomes (discussed in Sec. 2). Using polarization-sensitive OCT melanin has been shown to depolarize light.^149^ However, there has been little research into the effect of skin color on optical imaging applications using polarized light and this may warrant further study when used to detect skin cancers in patients with varying FSTs.

Others agree that there is huge potential for skin optical property inaccuracies due to melanin content and that more work in this area is required. Ray et al.^10^ urged researchers to increase subject diversity and sample sizes so that there is proportional representation.^10^ Colvonen^146^ argued that researchers should work together to raise standards in study quality and accurate reporting of the effect of skin color on wearable devices so as to close the racial healthcare bias.^146^ We would argue that the same should be applied to all optical healthcare applications that interact with the skin. Colvonen has suggested the following steps:^144^^,^^146^

• decrease use of the subjective skin tone measures;
• increase sample sizes to allow for interaction effects on skin tone;
• directly work with optical companies to advance their technology using multiple wavelengths for varying skin tones and to improve their effectiveness;
• hold the research community accountable for addressing and reporting bias;
• ensure that people of varying skin tones are included in validation and effectiveness research.

The headline results from our analysis of published data are described in Fig. 2. Briefly, absorption and reduced scattering coefficients are greater for FST I–II than FST V–VI between 400 and 1000 nm and 600 and 1000 nm, respectively; published coefficient variability within FST groups is large; there is limited data beyond 1000 nm; and most importantly, wavelengths beyond 940 nm are likely to be most useful for optical applications for all FSTs because TMFP and transmission are greatest in this spectral region. Because the published data beyond 1000 nm are minimal and not equivalently representative of all FSTs conclusions are difficult to draw. However, the existing data suggest that transmission may be increased beyond 1000 nm. We would, therefore, add to Colvonen’s list and state that studying an increased wavelength range is important as the current data suggest that the optical properties associated with different skin colors are likely to enable optimal transmission of light through the skin beyond 1000 nm. With increasing wavelengths, it is possible that light transmission through the skin will become independent of skin color enabling removal of the racial bias currently associated with optical medical technologies, this is something that should be investigated further. Therefore, a dataset that includes the optical properties of the skin for an equivalent range of FSTs between 400 and 1800 nm is required to fully understand the effect of wavelength with skin color and existing skin chromophores.

A clearer understanding of FST and the effect of the optical properties of skin is required to enable deeper imaging and consistent therapeutics without racial bias. A dataset of optical properties across a broad spectrum and in a large cohort of subjects with skin colors being accurately and equally represented is required. However, in so doing, multiple other variables must also be considered including subject age, gender, anatomical location, photo-exposure of the skin to light (i.e., tanning), body mass index, subject hydration, as well as measurement method.

## Data Availability

The data used in this review have been extracted from the open literature as referenced. The extracted data can be made available on reasonable request.
